# New Functionalized Polymeric Sensor Based NiO/MgO Nanocomposite for Potentiometric Determination of Doxorubicin Hydrochloride in Commercial Injections and Human Plasma

**DOI:** 10.3390/polym12123066

**Published:** 2020-12-21

**Authors:** Nawal A. Alarfaj, Maha F. El-Tohamy

**Affiliations:** Department of Chemistry, College of Science, King Saud University, P.O. Box 22452, Riyadh 11495, Saudi Arabia; nalarfaj@ksu.edu.sa

**Keywords:** doxorubicin hydrochloride, modified sensors, metal oxide nanocomposites, human plasma, commercial injections

## Abstract

The ultra-functional potential of nickel oxide (NiO) and magnesium oxide (MgO) nanoparticles (NPs), provides for extensive attention in the use of these metal oxides as a remarkable and electroactive nanocomposite in potentiometric and sensing investigations. This work proposed a new strategy for quantifying doxorubicin hydrochloride (DOX) in pharmaceuticals and human plasma by preparing a NiO/MgO core-shell nanocomposite modified coated wire membrane sensor. Doxorubicin hydrochloride was incorporated with phosphomolybdic acid (PMA) to produce doxorubicin hydrochloride phosphomolybdate (DOX-PM) as an electroactive material in the presence of polymeric high molecular weight poly vinyl chloride (PVC) and solvent mediator *o*-nitrophenyloctyl ether (*o*-NPOE). The modified sensor exhibited ultra sensitivity and high selectivity for the detection and quantification of doxorubicin hydrochloride with a linear relationship in the range of 1.0 × 10^−11^–1.0 × 10^−2^ mol L^−1^. The equation of regression was estimated to be E_mV_ = (57.86 ± 0.8) log [DOX] + 723.19. However, the conventional type DOX-PM showed a potential response over a concentration range of 1.0 × 10^−6^–1.0 × 10^−2^ mol L^−1^ and a regression equation of E_mV_ = (52.92 ± 0.5) log [DOX] + 453.42. The suggested sensors were successfully used in the determination of doxorubicin hydrochloride in commercial injections and human plasma.

## 1. Introduction

Advances in nanomaterial integration and nanoengineering technologies have opened new directions in research and development of modified sensor systems. The recent studies have shifted towards the formation of hybrid nanoparticles instead of single nanoparticles. These hybrid nanomaterials usually have two or more nanoscale domains, which cause synergistic potentials due to their interfacial interactions [1]. Nanocomposite materials possess both polymer advantages such as elasticity, biocompatibility, chemical resistance, and flexibility and the unique features of nanoparticles [1]. The current progress in scientific fields, such as materials science, engineering, physics, chemistry, and biology [2,3,4,5,6], require the development of sensing technologies that combine miniaturization with highly tactile sensitivity and low power consumption. Nanocomposites, are also materials with high potentials display unusual characteristic combinations and unique design possibilities. With an elevated annual growth rate and rapid demand to be in sensor fabrications, their potential is so striking that they are useful in various sensing applications [7]. They have emerged as possible alternatives to overcome the limitations of micro composites. Furthermore, they are considered to be the materials of the future, possessing unique design and advanced optical properties that are not found in conventional composites [8]. Additionally, the synthesis of nanocomposites is considered to be the vital key in bio and immunosensing detection, fabrication of electronics devices, medicine, drug delivery systems, and chemotherapy for cancer [9,10,11,12,13]. Recently, more studies are focused on the metal oxides such as zinc oxide, nickel oxide, magnesium oxide, etc. Furthermore, considerable attention has been focused on the use of nickel oxide nanoparticles (NiONPs) in various applications such as gas sensors, magnetic materials, catalysis, and electrocatalytic films [14,15,16,17]. Another important metal oxide is magnesium oxide nanoparticles (MgONPs) which is a very interesting alkaline oxide that has a high specific surface area with unique optical, catalytic, mechanical, and chemical properties [18,19,20,21]. The formation of a metal oxide nanocomposite protects the interaction of non-noble with the basic medium, and also coats the sensor wire to improve the substrate’s catalytic properties [22]. Therefore, NiO/MgO nanocomposite has been extensively used in catalysis. The multi-functional physicochemical potentials, large surface area, and powerful binding properties with high isoelectric stability of NiONPs with MgONPs, encourage the use of their nanocomposite in electrochemical sensors [23]. Many reports have addressed the preparation of NiO and MgO nanomaterials by different methods, including sol-gel, laser, thermal decomposition, and solvo-thermal technique [24,25,26,27,28,29]. An ion beam-assisted deposition method was reported to prepare NiO/MgO thin films for chemical transformations and catalytic applications [22].

Electrochemical techniques, including potentiometry, conductometry, and amperometry, have found a number of interesting applications in the scientific fields of clinical diagnosis, environmental, chemical analysis, and biomedical applications.

The potentiometric technique is one of the most promising electrochemical techniques that is known as a self-powered technique. The potentiometric measurements in self-powered sensors are conducted by the accumulation of analytes under the electrostatic mechanism leading to the formation of a potential difference between the surface of the working electrode and the reference one [30]. Potentiometric sensors with polymeric membrane containing electroactive material (ionophores or ion-pairs) can determine various dozens of compounds. The most commonly used materials in the fabrication of their membranes are high molecular weights of polyvinyl chloride (PVC), organic acids esters usually used as plasticizers such as dibutyl sebacate (DBS), dibutylphthalate (DBP), dioctyl sebacate (DOS), dioctylphthalate (DOP), etc. Furthermore, ether, e.g., *o*-nitrophenyloctyl ether (*o*-NPOE), is acting as the solvent mediator for ion-pairs. The electroactive materials are lipophilic ions or molecules able to induce specific interaction with other analyte ions in the membrane, which pre-detect the selectivity of the corresponding sensor [31]. Phosphomolybdic acid (PMA) or dodeca molybdophosphoric acid is a yellowish-green chemical compound that is freely soluble in polar solvents and water. It is commonly used in potentiometric studies due to its ability to react with various analytes [32,33].

In coated wire membrane sensor types, a highly conductive metal wire (Al, Cu, Pt, Au, or Ag) is used as a substrate and the polymer membrane cocktail coats the surface of the selected wire. Theses sensors are simple in shape, reproducible, provide fast potential response, mechanically stable, and usually exhibit excellent selectivity rather than the corresponding conventional liquid membrane type [34].

Doxorubicin hydrochloride (DOX) is a cancer chemotherapy medication often recommended under the brand name Adriamycin in combination with other medications to treat breast, bladder, lymphatic leukemia, and Kaposi’s sarcoma [35]. DOX was previously determined by various analytical methods, including separation techniques such as liquid chromatography [36], liquid chromatography coupled with tandem mass spectrometry [37], and capillary zone electrophoresis [38]. Spectrophotometric methods were also developed for the quantification of DOX [39]. Voltammetry was also reported for the determination of DOX [40]. However, only one potentiometric titration method was previously reported for the determination of DOX [41]. These reported techniques displayed good sensitivity for the determination of DOX, but still possess some drawbacks as they need long analytical times, high-skilled analysts, and consumption of large quantities of solvents.

The aim of this study is to fabricate an ultrasensitive and selective modified coated wire sensor enriched with electroactive NiO/MgO nanocomposite. The fabricated sensor was prospecting for potentiometric quantification of the breast cancer medication DOX in its pharmaceuticals and human plasma. The suggested method was validation to ensure the suitability of the modified sensor. Additionally, a comparative study was performed between the enriched NiO/MgO nanocomposite membrane sensor and the conventional fabricated type.

## 2. Experimental

### 2.1. Chemicals and Reagents

Pure grade of breast cancer medication doxorubicin hydrochloride and Adriamycin^®^ injections (50 mg doxorubicin hydrochloride/25 mL) was supplied by Pfizer, Saudi Co. Ltd. (Jeddah, Saudi Arabia). Different chemicals and solvents, such as ethanol 99.9%, methanol 99.9%, acetone 99.9%, ortho-nitrophenyloctyl ether (*o*-NPOE), hydrochloric acid 37%, tetrahydrofuran (THF) 97.0%, phosphomolybdic acid (PMA), and high molecular weight polyvinyl chloride (PVC) were obtained from Sigma Aldrich (Hamburg, Germany). Magnesium nitrate, citric acid, nickel nitrate, and sodium hydroxide were supplied by BDH (Poole, UK). Real blood samples were collected from patients in King Khalid hospitals (Riyadh, Saudi Arabia). The informed consent was approved for all volunteers before starting this work and the research ethics committee at King Saud University; Riyadh, Saudi Arabia (KSU-REC-002-E, 2020) approved the study.

### 2.2. Instruments

All potentiometric measurements were performed using a digital pH meter HANNA, model 211 (HANNA instruments, Rhode Island, Woonsocket, USA). To control the pH conditions of the tested solutions, Metrohm pH-meter model 744 (Metrohm Co., Herisau, Switzerland) was used. The potentiometric system consists of a fabricated conventional doxorubicin hydrochloride phosphomolybdate (DOX-PM) or modified DOX-PM-NiO/MgO nanocomposite coated wire sensors in connection with a reference electrode—silver/silver chloride (Ag/AgCl). The spectroscopic characterization was carried out using a spectrophotometer (Shimadzu Corporation, Kyoto, Japan), X-ray diffraction (XRD) (Shimadzu XRD-6000 diffractometer, Kyoto, Japan), and Fourier-Transform Infrared spectroscopy (FT-IR) Spectrum BX spectrometer, (PerkinElmer, Waltham, USA). However, microscopic detection was performed using a scanning electron microscope (SEM) (JSM-7610F; Akishima, Tokyo, Japan) and a transmission electron microscope (TEM) (JEM-2100F, JEOL Ltd., Akishima, Tokyo, Japan). Energy-Dispersive X-Ray Spectroscopy (EDX) analysis was performed using a SEM microscope in connection with EDX to confirm the presence of Ni and Mg elements in the prepared samples.

### 2.3. Synthesis of Magnesium Oxide Nanoparticles

MgONPs were typically prepared by adding 10 mL 0.02 mol L^−1^ of citric acid to 50 mL of aqueous 0.01 mol L^−1^ Mg(NO_3_)_2_ for 10 min. Under magnetic stirring, the solution was heated to 100 °C for 30 min. A gel solution was obtained. Then, it was heated in oven at 180 °C. The gel was dried to provide a fluffy powder, the precursor of MgO. The formed MgO was calcined at 800 °C to get MgO nanocrystals [42].

### 2.4. Synthesis of Nickel Oxide/Magnesium Oxide Nanocomposite

**Figure 1:**
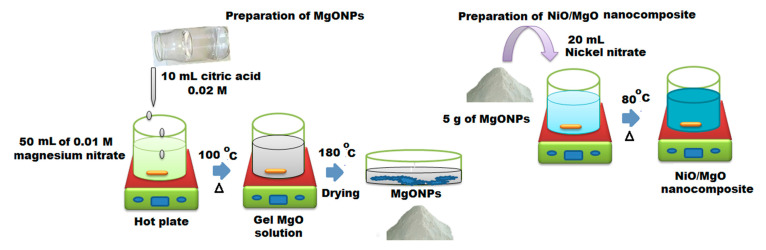
Schematic steps for the reparation of magnesium oxide nanoparticles (MgONPs) and NiO/MgO nanocomposite.

The preparation of NiO/MgO nanocomposite was performed by mixing 0.5 g of previously synthesized MgONPs with a 20 mL ethanolic solution of 0.01 mol L^−1^ nickel nitrate Ni(NO_3_)_2_·6H_2_O, under magnetic stirring for 30 min, for 12 h at 80 °C and then, calcined at 600 °C in the open air of a furnace for 2 h [42]. The preparation steps of MgOMPs and NiO/MgO nanocomposite were demonstrated in Figure 1.

### 2.5. Characterization of Nanoparticles

Spectroscopic detection was carried out using a (UV 2450 Spectrophotometer, Shimadzu, Kyoto, Japan) at a wavelength range of 200–500 nm to confirm the formation of NiO and MgO nanoparticles. FT-IR detection was also performed using a (PerkinElmer, Waltham, MA, USA) to estimate the possible functional groups that can present in the synthesized NiONPs and MgONPs. XRD analysis was performed using a (Shimadzu XRD-6000 diffractometer, Kyoto, Japan) using Kα radiation (*λ* = 1.5418 Å) under an operating current of 35 mA and a voltage of 40 kV. XRD outcomes were measured at a scan rate of 0.3 s/point and 0.02° resolution at room temperature. The surface morphology, shape, and size distribution were determined under TEM and SEM.

### 2.6. Preparation of Stock Drug Solution

A standard 1.0 × 10^−2^ mol L^−1^ DOX solution was prepared by dissolving 0.58 g of DOX in 100 mL distilled water. The solutions for analysis were prepared by carrying out various dilutions using distilled water.

### 2.7. Preparation of Electroactive Complex

The electroactive complex DOX-PM was obtained by adding 50 mL of an aqueous DOX solution (1.0 × 10^−2^ mol L^−1^) to the same volume of PMA solution (1.0 × 10^−2^ mol L^−1^). A yellow precipitate of DOX-PM was obtained. The precipitate formed was filtered and washed thoroughly using distilled water and left to dry at 25 °C for 24 h.

### 2.8. Membrane Composition and Sensor Fabrication

**Figure 2:**
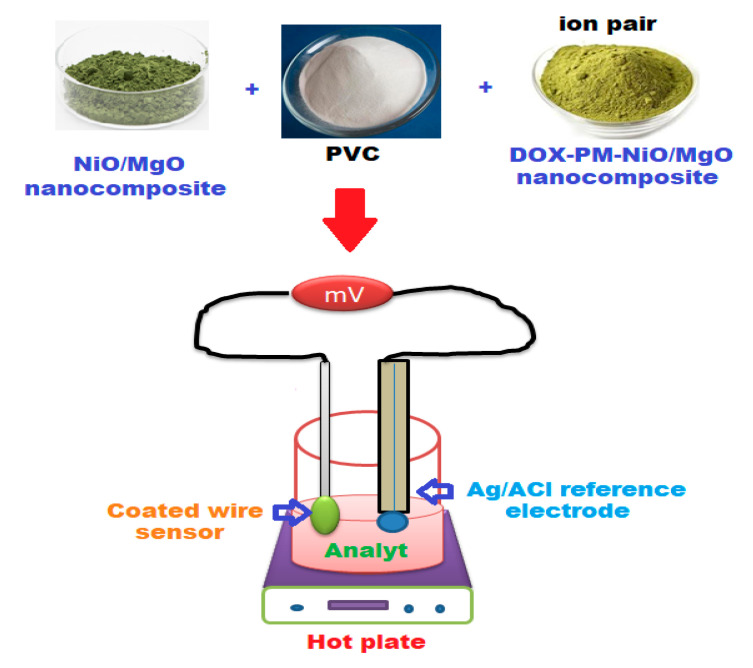
Schematic diagram for the preparation of the modified coated wire membrane sensor and the potentiometric system.

Two different coated membranes were prepared using electroactive materials DOX-PM and DOX-PM-NiO/MgO nanocomposite. The conventional coated wire membrane was prepared by mixing (PVC, 190 mg), with the electroactive material (DOX-PM, 10 mg) and plasticizer (*o*-NPOE, 0.35 mL) in 5 mL of THF. The obtained mixture was poured in a Petri dish (3 cm in diameter) and allowed to evaporate slowly at the ambient temperature. To prepare the modified membrane, 5 mg of the previously prepared NiO/MgO nanocomposite was added to the above-mentioned membrane composition. After cleaning and drying the tip of two aluminum wires with distilled water and acetone, sensor fabrication was conducted by immersing the cleaned wires several times in the membrane solution. The coated wire sensors were assembled as: Al wire/coated membrane/test solution//Ag/AgCl reference electrode. Figure 2 illustrated the preparation of the polymeric membrane cocktail and the construction of potentiometric system.

### 2.9. Calibration Graphs

The calibration graphs of both conventional and modified coated wire DOX-PM and DOX-PM-NiO/MgO nanocomposite sensors were graphed by plotting the potential responses of 50 mL of DOX standard solution in the concentration range 1.0 × 10^−11^–1.0 × 10^−2^ mol L^−1^ using the fabricated working sensors separately in connection with a reference electrode (Ag/AgCl). The calibration graph of each sensor was plotted using the potential readings as a function of −logarithm DOX concentrations. Prior to every measurement, the surface of the sensors should be carefully cleaned with distilled water and dried with tissue paper.

### 2.10. Optimization of Potential Readings Conditions

The potential readings of the suggested sensors can be greatly influenced by the change of test solution pH. To determine the suitable pH range of the fabricated sensors, 1.0 × 10^−4^ mol L^−1^ of DOX test solution was acidified using 0.1 mol L^−1^ hydrochloric acid to decrease the pH value to less than 2. The potential readings were also measured after elevating the pH of the investigating solution using 0.1 mol L^−1^ sodium hydroxide. The pH graphs were graphed by plotting the potential response of the fabricated sensors vs. the pH values.

The selectivity of the fabricated conventional and modified nanocomposite sensors was tested using a separate solution method [43].

The potentiometric selectivity coefficient of each sensor was evaluated by preparing separate solutions of each 1.0 × 10^−3^ mol L^−1^ DOX and interfering species. The selectivity of conventional (DOX-PM) and modified (DOX-PM-NiO/MgO) nanocomposite sensors was measured in the presence of various possible interfering species such as cations (Na^+^, K^+^, Ag^+^, Ni^2+^, Cu^2+^, Zn^2+^, Mg^2+^, and Fe^3+^), sugars (lactose and glucose), and amino acids (L-histidine, ornithine, and glycine). The selectivity coefficient was calculated using the following equation:

Log K^pot^ = (E_2_ − E_1_)/S + Log [Drug] − Log [B^z+^]^1/z^ (1)

where K^pot^, E_1_, E_2_, B^z+^, and S are the selectivity coefficient, the electro potential of 1.0 × 10^−3^ mol L^−1^ DOX solution, the electrode potential of 1.0 × 10^−3^ mol L^−1^ of interfering species, interfering ions, and slope of the calibration graph, respectively.

The dynamic response time was investigated by measuring the potential response of the tested drug using the concentration range of 1.0 × 10^−11^–1.0 × 10^−2^ mol L^−1^.

### 2.11. Analysis of DOX in Adriamycin^®^ Injections

The content of two Adriamycin^®^ injections (50 mg/25 mL) were mixed well and an accurate amount 0.58 g was used to prepare 1.0 × 10^−2^ mol L^−1^ standard DOX solution that was dissolved in 100 mL distilled water. Serial dilutions were performed to obtain various concentrations of DOX within the range of 1.0 × 10^−11^–1.0 × 10^−2^ mol L^−1^. The suggested sensors DOX-PM and DOX-PM-NiO/MgO nanocomposite were separately used to determine the tested drug in its commercial injections.

### 2.12. Analysis of DOX in Real Human Plasma Samples

The concentration of DOX in human plasma samples was measured by, collecting 5 mL of blood samples after 0.25–240 h administration of drug from a forearm vein into vacuum heparinized tubes. The plasma was separated by centrifuging the sample for 10 min at 2500 rpm and a lower temperature of 10 °C. The samples were kept in an ice-water bath before and during the separation process. The fabricated modified sensor was used to analyze the concentrations of DOX in the prepared plasma samples. A regression equation was used to calculate the percentage recoveries.

## 3. Results and Discussion

### 3.1. Characterization of NiO/MgO Nanocomposite

**Figure 3:**
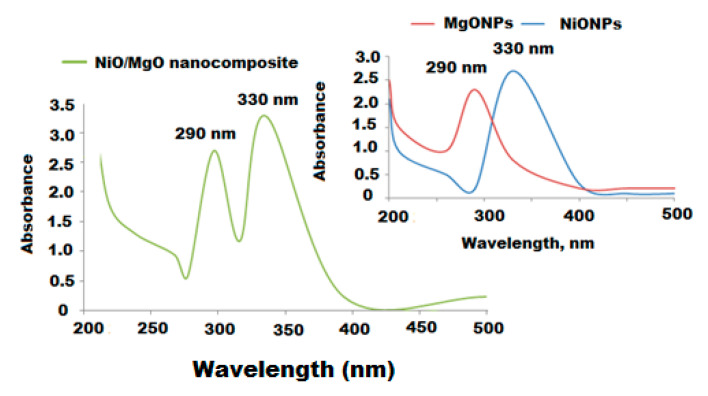
UV-Vis spectrum of the synthesized NiO/MgO nanocomposite with respect to MgONPs and NiONPs at an absorbance of 200–500 nm.

The prepared NiONPs/MgONPs were characterized using different spectroscopic methods including, UV-Vis, FT-IR, XRD, and EDX. UV–Vis spectroscopy was applied as a useful technique suitable for confirming the primary recognition of shape, size, and stability of the formed metal oxide nanoparticles in their aqueous suspensions. The UV-Vis spectrum of NiO/MgO nanocomposite showed two broad absorption peaks at 290 and 330 nm, respectively (Figure 3).

**Figure 4:**
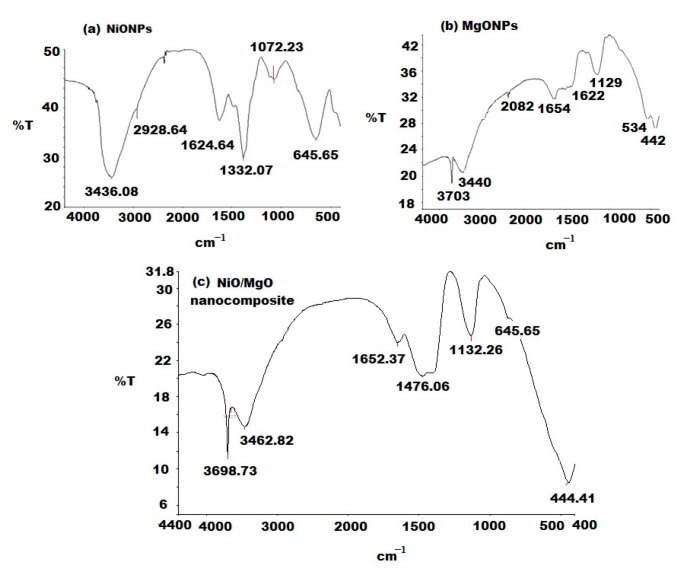
Fourier-Transform Infrared (FT-IR) spectra of the synthesized (**c**) NiO/MgO nanocomposite with respect to (**a**) NiONPs and (**b**) MgONPs at a wavenumber range from 4400 to 400 cm^−1.^

FTIR analysis for NiO/MgO nanocomposite with respect to the FTIR of NiONPs and MgONPs was performed in the range of 4400–400 cm^−1^. Different absorption bands of NiO/MgO nanocomposite were observed at 3698.73, 3462.82, 1652.37, 1476.06, 1132.26, 645.65, and 444.41 cm^−1^. The observed vibration bands at 3698.73 and 3462.82 cm^−1^ are related to O–H bond-stretching vibration. The absorption band that appeared at 1652.37 cm^−1^ revealed the presence of an O–H stretching mode of water. A recorded strong peaks at 1476.06 and 1132.26 cm^−1^ are attributed to CO_2_ of the surrounding atmosphere. The noticed peaks at 645.65 and 444.41 cm^−1^ confirmed the formation of Ni–O/Mg–O stretching vibrations (Figure 4c). The obtained results agree with those of single NiONPs and MgONPs with a slight shift and sharpness as shown in (Figure 4a,b).

**Figure 5:**
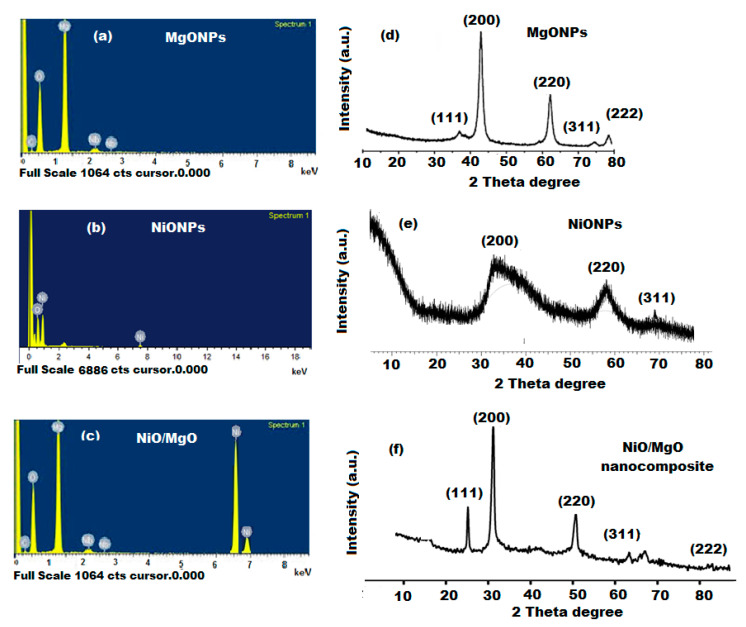
(**a**–**c**) Energy-Dispersive X-Ray Spectroscopy (EDX) and (**d**–**f**) X-Ray Diffraction (XRD) spectra of NiONPs, MgONPs, and NiO/MgO nanocomposite.

The EDX profile of NiO/MgO nanocomposite was detected using SEM equipped with an EDX spectroscopy and compared to the EDX profiles of NiONPs and MgONPs. The presence of Ni and Mg elements in NiO/MgO was evaluated. The recorded profiles indicated that the percentage elemental composition of Ni and Mg nanoparticles were 56.13% Ni and 43.86% O for NiONPs and 63.48% Mg and 36.52% O with a maximum peak intensity 1.2 keV and 1.5 keV for Ni and Mg, respectively (Figure 5a,b). However, the EDX profile of NiO/MgO nanocomposite showed 39.82% Ni, 42.4% Mg, and 17.72% O (Figure 5c) which confirmed the high purity of the as-prepared nanocomposite and the reduction of Ni and Mg ions to zero valences.

XRD analysis is one of the most useful spectroscopic techniques that is usually used to characterize the crystalline form of synthesized nanoparticles. The XRD pattern of NiO/MgO nanocomposite was recorded using an XRD diffractometer with Cu-kα at (k = 1.5405 Å). Sharp and well-defined peaks at 2θ values of 37.85, 41.98, 63.44, 74.83, and 78.00° corresponding to planes of (1 1 1), (2 0 0), (2 2 0), (3 1 1), and (2 2 2), respectively, were observed (Figure 5d,e). The obtained results could be indexed to face centered cubic structure with a lattice constant of a = 4.192 Å of NiO/MgO. These values matched well with the values of Joint Committee on Powder Diffraction Standards JCPDS No. 024-0712 of NiO/MgO bulk materials (Figure 5f).

**Figure 6:**
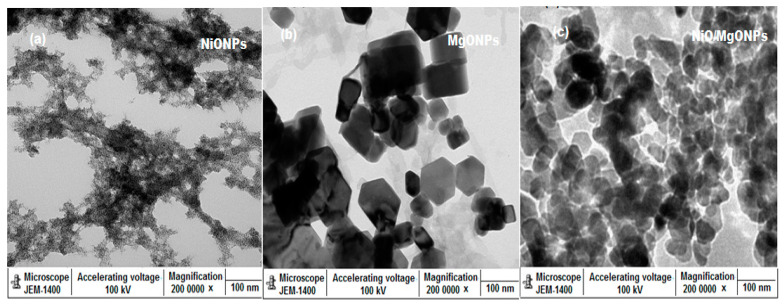
Transmission electron microscope (TEM) images of (**a**) NiONPs, (**b**) MgONPs, and (**c**) NiO/MgO nanocomposite at magnification 200,000×.

**Figure 7:**
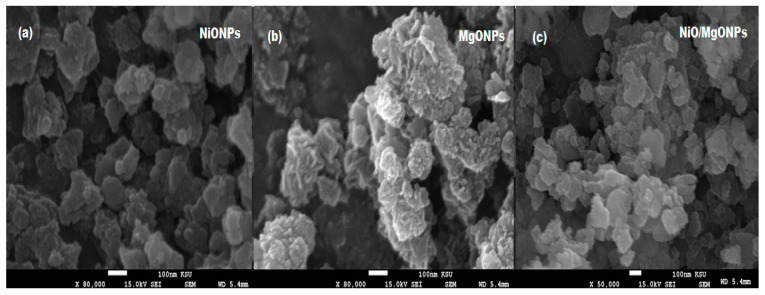
Scanning electron microscope (SEM) images of (**a**) NiONPs, (**b**) MgONPs, and (**c**) NiO/MgONPs.

Due to their very similar structure, not much difference is observed in the XRD patterns of NiO and NiO/MgO. Therefore, the formation of NiO/MgO can be identified using the above diffraction peaks. Further microscopic studies, using TEM and SEM, were performed to investigate the shape, size, and the surface morphology of the prepared NiO/MgO nanocomposite with respect to NiO and MgONPs. The obtained TEM (Figure 6a–c) and SEM (Figure 7a–c) images revealed uniformly distributed nanoparticles with spherical and hexagonal shapes for NiONPs and MgONPs, respectively, whereas NiO/MgO particle size was found to range from 80 to 100 nm with highly aggregated crystals in their surface morphology.

### 3.2. The Nature of the Fabricated Sensors

DOX reacts with PMA to form a stable DOX-PM complex, which is a stable complex that is soluble in organic solvents such as THF. The construction of conventional DOX-PM and modified coated wire DOX-PM-NiO/MgO nanocomposite sensors were carried out by mixing the illustrative materials with a solvent mediator (*o*-NPOE) and PVC in THF. In the present work, *o*-NPOE acts as a fluidizer helping the homogenous dissolution of the electroactive material and allowing for its diffusion mobility inside the membrane. The membrane selectivity was improved towards the tested analyte by the use of a high dielectric constant of *o*-NPOE (ε = 24), which affected the dissolution of ion pairs within the active membrane and consequently enhances its partition coefficient in the prepared membrane and provided a suitable mechanical feature for the membrane [44].

The potential response and the performance critical properties of the suggested DOX-PM and DOX-PM-NiO/MgO nanocomposite were summarized in Table 1.

**Figure 8:**
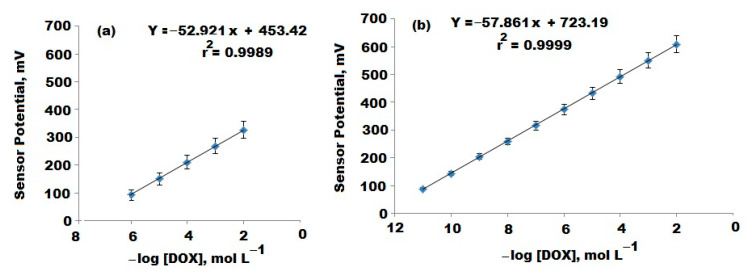
Calibration graphs of the fabricated (**a**) Conventional DOX-PM and (**b**) Modified DOX-PM-NiO/MgO nanocomposite coated wire sensors.

The results revealed that the above mentioned sensors displayed Nernstian responses with slopes of E_mV_ = (52.92 ± 0.5) log [DOX] + 453.42 and E_mV_ = (57.86 ± 0.8) log [DOX] + 723.19 mV over the DOX concentration ranges of 10 × 10^−6^–1.0 × 10^−2^ and 1.0 × 10^−11^–1.0 × 10^−2^ with correlation coefficients (0.9994 and 0.9999) for conventional DOX-PM and modified DOX-PM-NiO/MgO nanocomposite, respectively (Figure 8a,b).

The modified metal oxide DOX-PM-NiO/MgO nanocomposite sensor showed higher potentiometric response to a wider linear concentration range rather than the conventional one. The outcomes revealed a high sensitivity of the modified sensor towards the quantification of DOX, and that can be attributed to the large surface area of the coated nanoparticle layer which enhances the conductivity of the sensor surface. Furthermore, it was noticed that the use of nanocomposite provides higher results than the conventional type. This could be due to the high dielectric permittivity value of NiONPs (≈11.9) and MgONPs (≈3.2–9.8) at room temperature [45,46].

The fabricated conventional and modified sensors were tested with respect to dynamic response or the response to determine the time taken between the instant at which the potential of the cell becomes equal to its steady-state value within 1 mV. Under experimental conditions, including constant stirring and precondition of the sensors in the test sample, the potential readings were measured [47]. The measured dynamic response of a conventional DOX-PM sensor was found to be 75 s, whereas a modified DOX-PM-NiO/MgO nanocomposite showed a response time of 40 s. The enriched membrane with metal oxide nanocomposite displayed fastness and high mechanical stability compared to the conventional type. The presence of metal oxides with high surface area to volume ratios in the modified coated membrane and new physical and chemical features catalyzed the electrical conductivity of the modified sensor towards the interaction with the target analyte in the test solution. Additionally, the extraordinary electrical capacities and the exceptional electrical properties, including, the high charge transfer generated at the interfaces of some nanostructured materials, are of paramount importance when nanomaterials are used as the transducing components in potentiometric sensors [48].

**Figure 9:**
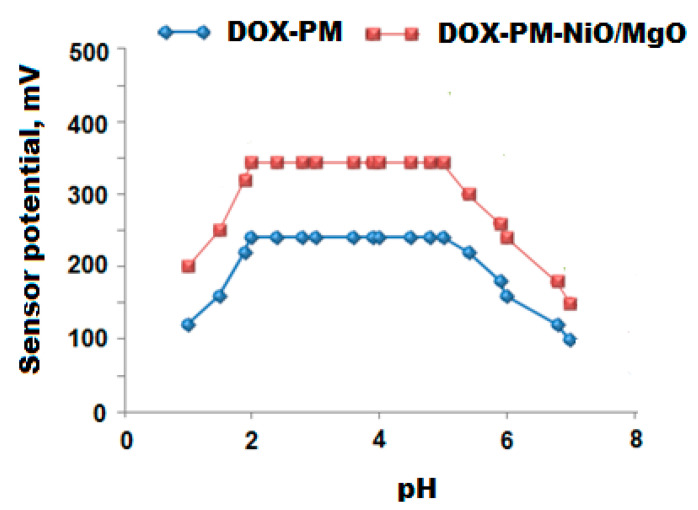
Effect of pH on the fabricated conventional DOX-PM and modified DOX-PM-NiO/MgO nanocomposite coated wire sensors using 1.0 × 10^−4^ mol L^−1^ of DOX solution.

The potential reading of the membrane sensor can be greatly affected by the interference of hydrogen ions. Therefore, it is very important to determine the safe pH range where the potential reading of the sensor is not influenced by hydrogen ions. The results indicated that both conventional DX-PM and modified DOX-PM-NiO/MgO sensors are practically pH independent in the pH range 2–5 and DOX can be easily determined using the fabricated sensors within this pH range (Figure 9).

The recorded results can be explained as follows: at pH less than 2, the hydrogen ions increased in the test solution and the potential of the sensors were slightly increased due to the formation of a protonated ion-pair that is poorly responsive to DOX ions. However, at pH values higher than 5 the potential readings were decreased gradually due to the increase of OH^−^ ions which causes a competition between DOX ions and OH^−^ ions and consequently decreases the interaction between the ions of the testing drug and the ion-pair sites on the sensor membrane. Thus, the potential responses of the constructed sensors were decreased [49].

Separate solution method [43] was applied to determine the selectivity coefficient of the fabricated conventional and modified sensors towards the determination of DOX in 1.0 × 10^−3^ mol L^−1^ of DOX and interfering species. The fabricated modified DOX-PM-NiO/MgO sensors showed excellent selectivity. The large surface area and physicochemical properties of metal oxide nanoparticles increases the conductivity of the fabricated sensor and hence increases its selectivity towards the tested DOX ions. Additionally, the selectivity of DOX coated membrane is due to free energy transfer of ions (DOX^+^) initiated between the active sites in the membrane and the testing solution. The obtained results indicated that no interference was observed by sugars and amino acids. Also, the difference of ionic size of the inorganic cations, their mobility, and permeability as compared with DOX^+^ prevents the interference of these cations during the analysis of the drug. Moreover, the smaller the energy of cation hydration, the greater response of coated membrane that is caused. Therefore, excellent selectivity and good tolerance were achieved by applying the DOX-PM-NiO/MgONPs sensor for the determination of DOX (Table 2).

### 3.3. Quantification of DOX in Its Bulk Powder

The fabricated conventional DOX-PM and DOX-PM-NiO/MgONPs sensors were applied to determine DOX in its bulk powder and the percentage recoveries were 98.8 ± 0.5 and 99.6 ± 0.4% for DOX-PM and DOX-PM-NiO/MgO nanocomposite, respectively (Table 3). The high sensitivity of the modified DOX-PM-NiO/MgO nanocomposite sensor was attributed to the unique physical and chemical features of the added metal oxide nanoparticles which causes an excellent sensitivity and selectivity towards the investigated drug, which was due to the high dielectric constant of the NiO and MgO nanocomposite.

### 3.4. Method Validation

The suggested potentiometric method was validated according to the guidelines of**** International Council for Harmonisation of Technical Requirements for Pharmaceuticals for Human ICH [50]. Two linear concentration ranges were displayed by the fabricated conventional DOX-PM and modified DOX-PM-NiO/MgO nanocomposite sensors with least square regression equations E_mV_ = (52.92 ± 0.5) log [DOX] + 453.42 and E_mV_ = (57.86 ± 0.8) log [DOX] + 723.19 with correlation coefficients 0.9994 and 0.9999 for the conventional DOX-PM and modified DOX-PM-NiO/MgO nanocomposite sensors, respectively. The lower limit of detection (LOD) was determined by measuring the potential readings of the fabricated sensors after the slope of each sensor was dropped by 17.9 mV. The outcomes showed LODs of 5.0 × 10^−7^ and 5.4 × 10^−12^ mol L^−1^ for conventional and modified sensors, respectively.

Nine DOX concentrations were used to study the accuracy of the developed method and the mean percentage recoveries were calculated as 99.2 ± 0.6% and 99.8 ± 0.2% for DOX-PM and DOX-PM-NiO/MgO nanocomposite, respectively. Also, the intra-day and inter-day assays were applied to confirm the precision of the suggested potentiometric method. The results were expressed as a percentage relative standard deviation (% RSD). The fabricated DOX-PM-NiO/MgONPs sensor showed 0.2% for both intra-day and inter day, respectively. All results are less than 2%, indicating a highly precise method (Table 4).

The method robustness was studied by inducing a slight change in the pH values of the tested solutions using acetate buffer pH 5 ± 0.5 and the obtained percentage recoveries were calculated and were found to be 98.7 ± 0.8% and 99.6 ± 0.2% for DOX-PM and DOX-PM-NiO/MgO nanocomposite sensors, respectively (Table 1). Further investigation was performed to evaluate the ruggedness of the suggested method by using another model of pH meter, Metrohm model 744. The results were represented as mean percentage recoveries which were found to be 99.0 ± 0.8% and 99.5 ± 0.3% for the conventional DOX-PM and modified DOX-PM-NiO/MgO nanocomposite sensors, respectively (Table 1). The results confirmed an excellent agreement with those obtained by the described method and no significant difference was observed.

### 3.5. Quantification of DOX in Adriamycin^®^ Injections

To quantify the breast cancer medication, DOX in its Adriamycin^®^ injections (50 mg/25 mL) the proposed method, using fabricated DOX-PM and modified DOX-PM-NiO/MgO nanocomposite sensors, was applied. The potential readings of the test solutions in the range of 1.0 × 10^−6^–1.0 × 10^−2^ and 1.0 × 10^−10^–1.0 × 10^−2^ mol L^−1^ were determined and the percentage recoveries were derived from the regression equations. The obtained results were found to be 99.3% ± 0.3% and 99.9% ± 0.2% for the above mentioned sensors, respectively (Table 5).

It was noticed that DOX-PM-NiO/MgO nanocomposite sensor exhibited ultra sensitivity towards the determination of DOX rather than DOX-PM and this was due to the high dielectric constant of NiO and MgO which increased the conductivity of the sensor and hence elevated the sensitivity. The outcomes were statistically analyzed using t-student’s and F-tests [51] and the results were compared with those obtained by the previously reported voltammetric method [40] which is based on determination of DOX using differential pulse cathodic stripping voltammetry on a polished silver solid amalgam electrode. The recorded results revealed an excellent sensitivity of the proposed sensors towards the determination of DOX in its injection solution.

**Table 5:** The outcomes from the determination of DOX in Adriamycin^®^ injections using fabricated DOX-PM and modified DOX-PM-NiO/MgO nanocomposite coated wire sensors in comparison with a reported method [41].

**Statistical Analysis**	**Conventional DOX-PM Coated Wire Sensor**		**Modified DOX-PM NiO/MgO Sensor**		**Reported** **Method** **[41]**
	* Test Solution	**%** **Recovery**	* Test **Solution**	**%** **Recovery**	
	**6**	**99.0**	**10**	**99.5**	**99.5 ± 0.4**
	**5.3**	**99.3**	**8**	**99.8**	
	**5**	**99.5**	**6**	**99.5**	
	**4**	**99.8**	**4**	**99.8**	
	**3**	**99.2**	**3**	**100.2**	
	**2**	**98.8**	**2**	**99.9**	
Mean ± SD	99.3 ± 0.3		99.8 ± 0.3		
*n*	6		6		
Variance	0.09		0.09		
%SE **	0.12		0.12		
%RSD	0.30		0.30		
*t*-test	1.000 (2.228) ***		1.500 (2.228) ***		
F-test	1.78 (5.05) ***		1.78 (5.05) ***		

* Test solution and Found using −log [DOX] mol L^−1^ ** SE (%Error) = %RSD/$\sqrt{n}$. *** The tabulated values of “*t*-test” and ‘‘F-test’’ at confidence level *p* = 0.05 [52].

### 3.6. Quantification of DOX in Human Plasma Samples

To prove the efficiency of the suggested modified DOX-PM-NiO/MgO nanocomposite sensor for the quantification of DOX breast cancer medication, further study was carried out using 15 plasma samples of patients prescribed to use DOX as breast cancer therapy. The suggested modifying sensor was used to analyze the real samples withdrawn from women ranging from 25 to 55 years old. The potential readings–concentration relationship was used to determine the quantity of the tested drug in three replicates after adding certain increments (0.5 mol L^−1^ of DOX) using the modified DOX-PM-NiO/MgO nanocomposite. The developed modified sensor showed excellent efficiency for the quantification of DOX with percentage recoveries (98.2–99.3%) and %RSD (0.4–1.4%). The outcomes were represented in Table 6.

A confirming study was performed by comparing the outcomes by other results obtained from a previously addressed method [52]. The random detection of plasma samples revealed that the modified DOX-PM-NiO/MgO nanocomposite sensor showed ultrasenstivity for the determination of DOX in bio samples rather than the previously reported method.

## 4. Conclusions

In this study, we have successfully fabricated a simply modified and highly sensitive potentiometric method NiO/MgO nanocomposite-based sensor. The fabricated sensor was prepared by modifying the coated membrane with NiO/MgO nanocomposite. The proposed modified nanosensor displayed immense surface area to volume ratios which provided excellent sensitivity. The results obtained by the modified metal oxide nanocomposite sensor for the determination of DOX were assessed statistically and compared to others of a conventional type. It was observed that the fabricated modified DOX-PM-NiO/MgO nanocomposite exhibited a higher potential response than the conventional sensor. Moreover, covering the sensor surface with a metal oxide nanocomposite layer increased the electroconductivity of this sensor and enhanced the determination of the investigated drug with high selectivity and sensitivity. Therefore, the fabrication of coated wire modified membrane sensor with metal oxide nanocomposite can be successfully applied for the quantification of DOX in commercial products and biofluids.

## Figures and Tables

**Table 1:** Performance characteristics of fabricated conventional coated wire doxorubicin hydrochloride phosphomolybdate (DOX-PM) and modified DOX-PM-NiO/MgONPs sensors.

Parameter	Conventional Coated Wire DOX-PM Sensor	Modified DOX-PM-NiO/MgO Nanocomposite Sensor
Slope (mV. Decade^−1^)	52.9 ± 0.5	57.9 ± 0.3
Intercept	453.4	723.2
Regression equation	E_mV_ = (52.9 ± 0.5) log [DOX] + 453.4	E_mV_ = (57.9 ± 0.3) log [DOX] + 723.2
Correlation coefficient, r	0.9989	0.9999
Linear range (mol L^−1^)	10 × 10^−6^–1.0 × 10^−2^	1.0 × 10^−11^–1.0 × 10^−2^
LOD	5.0 × 10^−7^	5.4 × 10^−12^
Response time/s	75	40
Working pH range	2–5	2–5
Lifetime/day	30	75
Temperature (°C)	25	25
Accuracy (%)	99.2 ± 0.6	99.8 ± 0.2

**Table 2:** Selectivity coefficient (K^Pot^_DOX_^+^) of the conventional coated wire DOX-PM sensor and the modified DOX-PM-Ni/MgO nanocomposite by the separate solution method using 1.0 × 10^−3^ mol L^−1^ DOX.

Interferences	Conventional Coated Wire DOX-PM Sensor $\left(K_{DOX^{+}}^{pot}\right)$	Modified DOX-PM-NiO/MgO Nanocomposite Sensor $\left(K_{DOX^{+}}^{pot}\right)$
Na^+^	4.4 × 10^−3^	9.5 × 10^−5^
K^+^	1.4 × 10^−3^	5.8 × 10^−4^
Ca^2+^	2.9 × 10^−3^	6.4 × 10^−4^
Mg^2+^	6.6 × 10^−3^	2.5 × 10^−5^
Cu^2+^	4.8 × 10^−3^	8.9 × 10^−4^
Zn^2+^	7.6 × 10^−3^	2.8 × 10^−5^
Ag^+^	6.9 × 10^−3^	5.9 × 10^−4^
Glucose	4.6 × 10^−3^	4.4 × 10^−5^
Lactose	5.9 × 10^−3^	5.7 × 10^−4^
Starch	4.4 × 10^−3^	2.3 × 10^−5^
Valine	1.3 × 10^−3^	9.9 × 10^−4^
Lysine	4.6 × 10^−3^	3.4 × 10^−5^
Tryptophan	9.5 × 10^−3^	5.9 × 10^−5^
Glycine	5.4 × 10^−3^	6.7 × 10^−5^
Leucine	1.6 × 10^−3^	2.6 × 10^−4^
L-histidine	4.7 × 10^−3^	2.8 × 10^−5^

**Table 3:** The outcomes from the determination of DOX in pure form using conventional fabricated DOX-PM and modified DOX-PM-NiO/MgO nanocomposite coated wire sensors.

**Statistical analysis**	**Conventional DOX-PM Coated Wire Sensor**		**Modified DOX-PM-NiO/MgO Nanocomposite**	
	* Test **solution**	**%** **Recovery**	* Test **Solution**	**%** **Recovery**
	**6** **5.3** **5** **4** **3** **2**	**98.5** **98.2** **99.4** **99.3** **98.8** **98.6**	**11** **10** **9** **8** **7** **6** **5** **4** **3** **2**	**100.0** **99.5** **99.7** **100.0** **98.9** **99.2** **99.6** **100.0** **99.6** **99.7**
Mean ± SD	98.8 ± 0.5		99.6 ± 0.4	
*n*	6		10	
Variance	0.25		0.16	
%SE	0.20		0.13	
%RSD	0.51		0.40	

* Test solution using −log [DOX] mol L^−1^.

**Table 4:** The precision assay of the suggested method using modified Modified DOX-PM-NiO@MgO nanocomposite coated wire sensor.

Statistical analysis	**Modified DOX-PM-NiO@MgO Nanocomposite Coated Wire Sensor**					
	Intra-day assay			Inter-day assay		
	* Test solution	* Found	% Recovery	* Test solution	* Found	% Recovery
	11.0	11.0	100.0	11.0	10.99	99.9
	8.0	7.99	99.9	8.0	7.98	99.8
	4.0	4.01	100.3	4.0	3.98	99.5
Mean ± SD	100.06 ± 0.2			99.7 ± 0.2		
*n*	3			3		
Variance	0.04			0.04		
%SE **	0.11			0.11		
%RSD	0.20			0.20		

* Test solution and Found using −log Conc. mol L^−1^, ** SE (%Error) = %RSD/$\sqrt{n}$.

**Table 6:** The results obtained from the determination of DOX in human plasma using modified DOX-PM-NiO/MgONPs coated wire sensor in comparison with a reported method [52].

Initial [DOX] −log Conc. mol L^−1^	Added [DOX] −log Conc. mol L^−1^	DOX-PM- NiO/MgONPs Sensor	Reported Method [52]
		% Recovery ± %RSD	% Recovery ± %RSD
8.3	0.5	98.4 ± 0.6	96.4 ± 0.5
6.5	0.5	99.0 ± 0.4	96.5 ± 0.7
8.8	0.5	98.2 ± 0.3	97.3 ± 0.8
7.6	0.5	99.5 ± 0.2	96.9 ± 0.4
8.2	0.5	98.9 ± 0.7	97.6 ± 0.2
5.9	0.5	98.4 ± 0.9	98.6 ± 1.4
7.7	0.5	99.2 ± 0.3	96.4 ± 1.2
9.7	0.5	99.6 ± 0.4	97.8 ± 0.7
6.2	0.5	98.5 ± 1.1	98.5 ± 0.4
8.6	0.5	98.8 ± 0.9	96.6 ± 1.2
7.2	0.5	98.6 ± 1.2	97.4 ± 0.3
6.1	0.5	98.3 ± 0.5	98.8 ± 14
8.7	0.5	98.2 ± 0.3	96.9 ± 0.5
7.8	0.5	99.3 ± 0.9	97.4 ± 0.6
7.4	0.5	98.6 ± 1.0	98.3 ± 0.3
8.6	0.5	99.8 ± 0.8	97.9 ± 1.4

## Data Availability

All data involved in this study included within the text.
